# Penta-Octa B_4_C_2_N_3_: A New 2D Material for High-Performance Energy Applications

**DOI:** 10.1021/acs.langmuir.4c05139

**Published:** 2025-02-22

**Authors:** Xihao Chen, Jiazhuo Wang, Nicolas F. Martins, Julio R. Sambrano, José A. S. Laranjeira

**Affiliations:** † School of Materials Science and Engineering, Chongqing University of Arts and Sciences, Chongqing 402160, China; ‡ School of Sciences, Modeling and Molecular Simulation Group, São Paulo State University (UNESP), Bauru, São Paulo 17033-360, Brazil

## Abstract

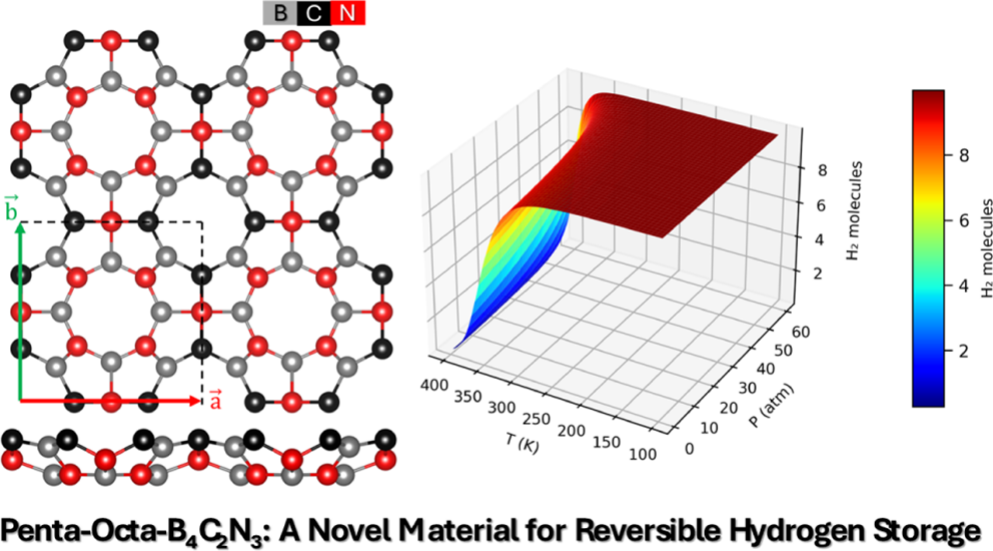

Penta-octagraphene (POG) is a newly suggested two-dimensional carbon allotrope recognized for its distinct configuration and fascinating electronic characteristics. This work presents a new inorganic counterpart of POG, named POG-B_4_C_2_N_3_, designed through density functional theory (DFT) calculations. This new structure exhibits a direct band gap transition at the X-point, measured at 0.32/0.86 eV with PBE/HSE functionals. Mechanical properties were comprehensively assessed, showcasing its Young’s modulus (*Y*_*max*_/*Y*_*min*_ = 157.12/100.84 N/m) and shear modulus (*G*_*max*_/*G*_*min*_ = 83.03/38.09 N/m), alongside Poisson’s ratio (ν_*max*_/ν_*min*_ = 0.58/-0.09), indicating that POG-B_4_C_2_N_3_ is an auxetic material. Additionally, Li decoration on this monolayer was studied to investigate its potential to enhance hydrogen storage through physisorption. The Li@POG-B_4_C_2_N_3_ system shows robust physisorption (adsorption energies ranging from −0.35 to −0.19 eV), high hydrogen storage capacity (8.35 wt %), and effective hydrogen desorption dynamics, positioning this novel material as a promising platform for reversible hydrogen storage.

## Introduction

The increasing level of environmental degradation has led to the need for technologies that facilitate the use of clean and renewable energy sources.^1−3^ Hydrogen (H_2_) emerges as a promising candidate due to its exceptionally high energy density of 142 MJ/kg and its environmentally friendly nature, which does not produce pollutants upon use.^4−6^ However, the primary challenge in the transition from fossil fuels to hydrogen lies in developing methods for safe and efficient storage. Guidelines from the U.S. Department of Energy (DOE) suggest that optimal storage systems should achieve a gravimetric capacity between 4.5 and 6.5 wt % and a volumetric density in the range of 40–50 g/L.^7^ Hydrogen storage can be achieved through various means, including gaseous or liquid states, as well as through solid-state mechanisms involving physisorption or chemisorption.^8−11^

Recent advances in material science have highlighted the potential of two-dimensional (2D) materials, such as graphene^12−14^ and MXenes,^15−17^ as promising candidates for hydrogen storage. In general, 2D materials exhibit reversibility and cyclic stability under ambient conditions, making them highly attractive for hydrogen storage applications.^18−21^ Many 2D materials also align with the performance criteria set by the DOE, including adsorption–desorption temperature, hydrogen uptake density, and operational pressure requirements.^22−29^

In this context, penta-octa-graphene (POG) is a recently proposed 2D carbon allotrope that stands out due to its unique structure and intriguing electronic properties.^30^ This structure has been predicted to exhibit both type-I and type-II Dirac line nodes (DLNs). Type-I DLNs arise from the inversion of the band between conduction and valence bands, while type-II DLNs are formed by interactions between the two highest valence bands. These DLNs represent topological states in which energy bands intersect along continuous lines in momentum space, providing pathways for unique electronic transport phenomena.

Penta-octa-graphene (POG) has shown remarkable potential for hydrogen storage^31^ and CO_2_ reduction (CO_2_RR)^32^ applications, as revealed by density functional theory studies. For hydrogen storage, POG decorated with alkali metals (Li, Na, and K), alkaline earth metals (Ca), and transition metals (Sc, Ti, V, Cr, and Mn) exhibits stable adsorption without clustering (except for Be and Mg). Notably, 4Li@POG and 2Ti@POG achieve ideal hydrogen adsorption energies (0.15–0.60 eV), facilitating reversible storage at ambient conditions. These configurations support up to 3 and 5 H_2_ molecules per Li and Ti atom, respectively, with gravimetric densities reaching 9.9 wt % (Li) and 6.5 wt % (Ti). In the CO_2_RR, POG and transition metal-decorated POG (TM@POG) serve as effective electrocatalysts, with Fe@POG outperforming Cu (211) in CH_4_ production. TM atoms enhance the catalytic performance by facilitating electron transfer to intermediate species, promoting CO_2_ activation, and reducing reaction complexity.

In addition, the POG lattice recently demonstrated potential as a host for inorganic compositions, with a silicon carbide-based derivative, POG-Si_5_C_4_.^33^ DFT simulations confirm its energetic viability, dynamical stability, and thermal resilience up to 1020 K. POG-Si_5_C_4_ is a semiconductor with a 2.02 eV indirect bandgap, making it suitable for electronic and optoelectronic devices. Its anisotropic mechanical properties include a Young’s modulus of 38.65–99.47 N/m and a negative Poisson’s ratio (−0.09). Additionally, its band edge alignment supports photocatalytic water splitting for hydrogen production, underscoring the potential of the POG lattice for advanced material designs in renewable energy.

This work introduces a novel inorganic analogue of POG, namely, POG-B_4_C_2_N_3_ via DFT simulations. The design of BCN-based monolayers is highly attractive due to the similar atomic radii of the constituent atoms (B, C, and N), which contribute to structural stability, and the remarkable electronic and mechanical properties arising from the tailored lattice composition.^34−41^ Furthermore, ternary compounds are particularly desirable for hydrogen storage applications because they offer numerous adsorption sites capable of retaining hydrogen in the gas phase. Notable examples include B_3_CN_4_,^42^ porous BCN,^43^ 4–10–16 BCN,^44^ and square-octagon BCN,^45^ all of which have been successfully demonstrated as promising hydrogen storage materials with theoretical hydrogen storage capacities of 10.70, 10.73, 6.43, and 11.66 wt %, respectively.

The stability of the novel POG-B_4_C_2_N_3_ compound was confirmed through key descriptors, including cohesive energy, phonon dispersion, Born-Huang criteria, and *ab initio* molecular dynamics simulations. POG-B_4_C_2_N_3_ is identified as a narrow band gap semiconductor with a direct band gap of 0.86 eV at the HSE level. Its mechanical properties were thoroughly investigated, highlighting Young’s modulus, shear modulus, and Poisson’s ratio. Additionally, Li decoration on this monolayer was explored as a strategy to enhance hydrogen storage via physisorption. This study is expected to inspire both theorists and experimentalists to explore POG-based structures further, contributing to the development of 2D materials with advanced properties for energy applications.

## Computational Setup

This study used the generalized gradient approximation (GGA)^46^ based on the Perdew, Burke and Ernzerhof (PBE)^47^ functional and the projector augmented wave (PAW) method,^48^ both implemented in the Vienna ab initio simulation package (VASP). A cutoff energy of 520 eV was utilized to represent the electronic states. To prevent interactions between periodic adjacent layers, a vacuum layer of 15 Å was fixed along the *c*-axis. A Γ-centered k-point grid of 5 × 5 × 1 was used for structural optimizations and partial density of states (PDOS) calculations. The DFT-D2 and D3 dispersion corrections proposed by Grimme were included. Structural optimization was performed using the conjugate gradient algorithm until the convergence criteria were met, requiring energy errors for atomic positions and lattice parameters to be less than 1 × 10^–5^ eV and Hellmann–Feynman forces on each atom to be within 0.01 eV/Å. To evaluate the thermodynamic stability of Na-decorated B_7_N_5_ and its hydrogen storage reversibility, ab initio molecular dynamics (AIMD) simulations were conducted. Additionally, the charge transfer was quantified through a Bader charge analysis.

The thermodynamic stability of the POG-B_4_C_2_N_3_ was assessed by computing its cohesive energy (*E*_coh_), which is defined by
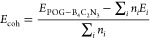
1where  is the total energy of the POG-B_4_C_2_N_3_ structure, *E*_*i*_ is the energy of an isolated atom *i* (B, C, and N), and *n*_*i*_ is the number of *i* atoms in the sheet.

The hydrogen adsorption energy (*E*_ads_) for the Li@POG-B_4_C_2_N_3_ + nH_2_ configurations is determined using the equation:
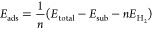
2where *E*_total_ is the total energy of the Li@POG-B_4_C_2_N_3_ + nH_2_ system, *E*_sub_ refers to the energy of the Li@POG-B_4_C_2_N_3_ substrate, and  is the energy of an isolated H_2_ molecule.

The hydrogen adsorption capacity (HAC) is defined as
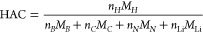
3where *n*_*X*_ and *M*_*X*_ are the number of atoms and molar masses of H, B, N, and Li, respectively.

The hydrogen release temperature (*T*_R_) is obtained using the van’t Hoff equation,^49,50^ considering atmospheric pressure (1 atm):
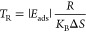
4where *R* and *k*_B_ are the universal gas constant and the Boltzmann constant, respectively, and Δ*S* is the change in entropy of hydrogen from the gas to liquid phase (75.44 J mol^–1^ K^–1^).

## Results and Discussion

### POG-B_4_C_2_N_3_ Monolayer

**Figure 1:**
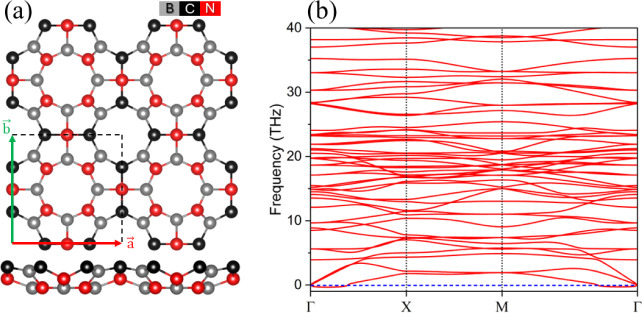
(a) POG-B_4_C_2_N_3_ lattice composed by octagonal rings BN and BC octagonal rings intercalated with B_2_N_2_C pentagonal rings and (b) phonon band dispersion for the new monolayer.

POG-B_4_C_2_N_3_ consists of a square unit cell with lattice parameters *a* = *b* = 6.784 Å, belonging to the *P*4*mm* (No. 99) space group and described by the C*_4v_* point group. This network comprises octagonal rings BN and BC intercalated with B_2_N_2_C pentagonal rings, as illustrated in Figure 1a. The structure contains five nonequivalent bond lengths: *l*_1_*(B–N)* = 1.41 Å, *l*_2_*(B–N)* = 1.61 Å, *l*_3_*(B–N)* = 1.50 Å, *l*_4_*(B–C)* = 1.51 Å, and *l*_5_*(C–N)* = 1.51 Å. Furthermore, a nonzero buckling height of 1.27 Å is observed, which is greater than that reported for POG (1.23 Å)^30^ but less than the value observed for POG-Si_5_C_4_ (2.07 Å).^33^

The thermodynamic stability of the monolayer was assessed by calculating its cohesive energy (*E*_coh_) by using eq 1. The result was −1.38 eV/atom, indicating that this monolayer remains stable relative to its ground-state atoms.

Figure 1b displays the phonon band dispersion for POG-B_4_C_2_N_3_ along the high-symmetry Brillouin zone pathway. It reveals minor negative frequencies near the Γ point, specifically, −0.33 THz (−11 cm^–1^), which are artifacts resulting from the supercell size used in the phonon band calculations. According to Wang et al.,^51^ such artifacts do not indicate the instability of this 2D material.

**Figure 2:**
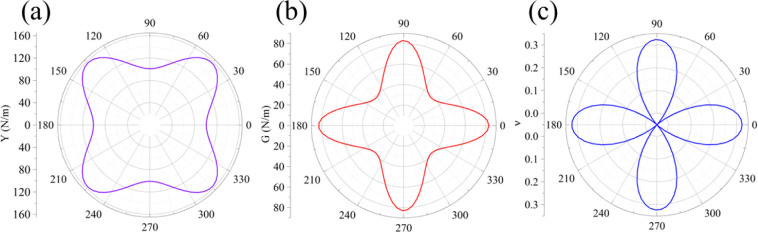
Polar representation of (a) Young’s modulus (Y), (b) shear modulus, and (c) Poisson’s ratio (ν) of the POG-B_4_C_2_N_3_ monolayer.

The mechanical property analysis shows that POG-B_4_C_2_N_3_ has elastic constants C_11_ = C_22_ = 112.64 N/m, C_12_ = C_21_ = 36.46 N/m, and C_66_ = 83.02 N/m, which satisfy the Born–Huang^52^ criteria for a square lattice, i.e., C_11_ > 0, C_66_ > 0, C_11_ > |C_12_|, indicating mechanical stability and, therefore, demonstrating the mechanical stability of this new monolayer. Figure 2 illustrates the spatial dependence of Young’s modulus (Y), shear modulus (G), and Poisson’s ratio (ν). It can be seen that all of the parameters present anisotropic behavior. For Y, maximum/minimum values of 157.12/100.84 N/m were found, which is higher than those obtained for POG-Si_5_C_4_ (99.47/38.65 N/m). The maximum/minimum values of G were found to be 83.03/38.09 N/m. The ν presents the most notable anisotropy with maximum/minimum values of 0.32/–0.05, which highlights POG-B_4_C_2_N_3_ as an auxetic material, a characteristic shared with its carbon (0.42/–0.10) and silicon carbide (0.58/–0.09) counterparts.^33^ The emergence of a negative Poisson’s ratio (NPR) in POG-B_4_C_2_N_3_ suggests an enhanced mechanical response of this monolayer under stress and greater effectiveness for energy dissipation. In addition, to achieve hydrogen storage applications, the novel POG-B_4_C_2_N_3_ can offer better management of volumetric expansion or contraction during H_2_ transport.

**Figure 3:**
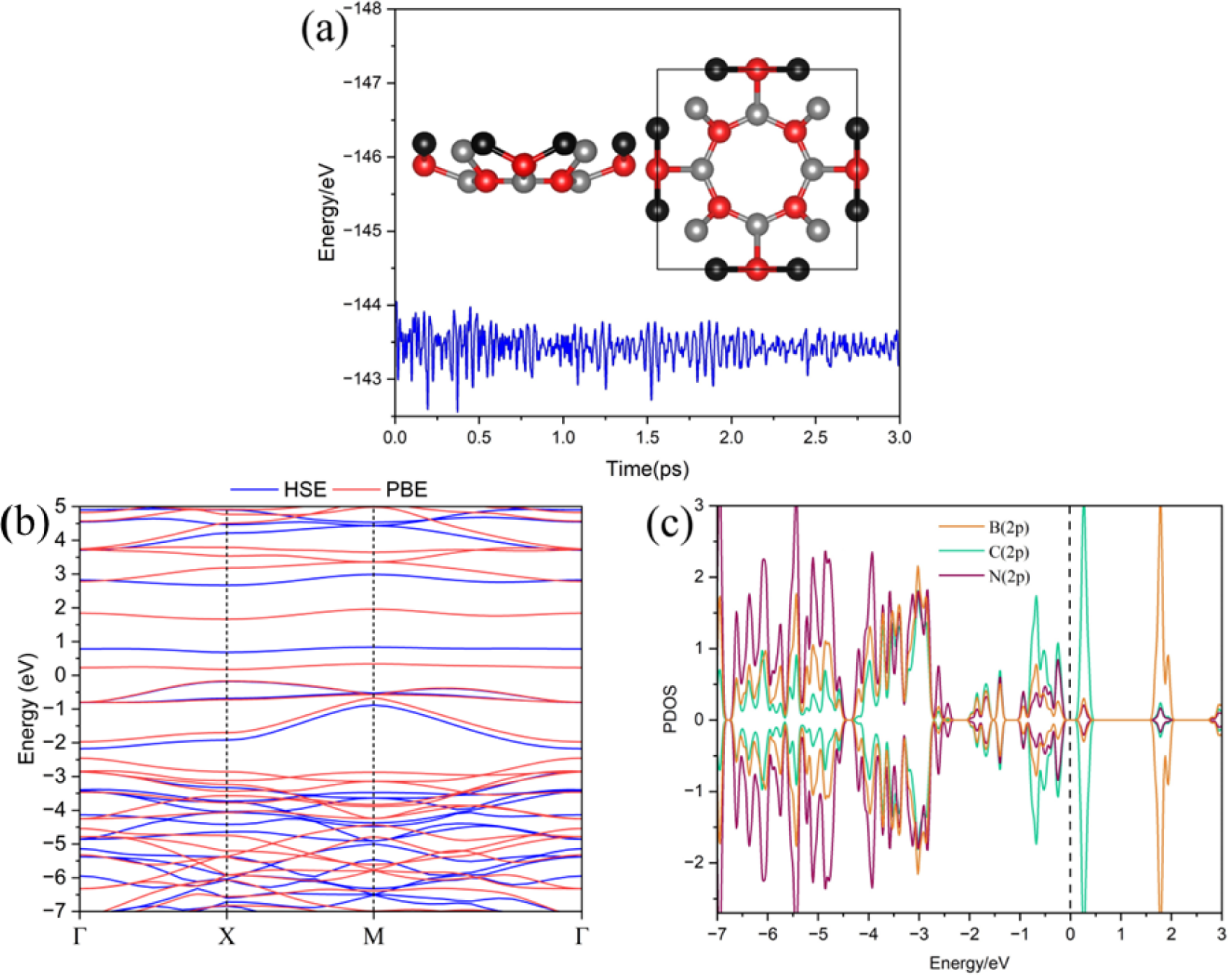
(a) Energy fluctuations and final structure obtained from AIMD simulations at 300 K, (b) band structure obtained at both PBE and HSE levels, and (c) partial density of states (PDOS) for the new POG-B_4_C_2_N_3_ nanosheet.

Figure 3a presents the energy fluctuations during the AIMD simulations at 300 K for the pristine POG-B_4_C_2_N_3_ monolayer, along with a snapshot from the last iteration. It is observed that no significant distortions or reconstructions were detected at the end of the simulation. The energy variation profile exhibits fluctuations below 1 eV/atom, indicating that the monolayer remains stable at room temperature. The band structure and partial density of states (PDOS) are shown in Figures 3b,c, respectively.

For the band structure, calculations were performed by using both the PBE (red) and HSE (blue) functional levels for comparison. PBE typically underestimates the bandgap energy, while HSE provides a more reliable description of the material’s bandgap. Both functionals reveal a direct band gap transition with the valence band maximum (VBM) and conduction band minimum (CBM) located at the X-point. The band gap energies (E_*gap*_) were found to be 0.32 eV for PBE and 0.86 eV for HSE.

Regarding the PDOS, it is observed that for lower energies within the valence band (VB), the B and N states dominate the distribution, while at the VBM, the major contribution comes from the C states. For the CBM, C states also play a significant role, and at higher conduction band energies, the B states become more pronounced. The flatness of the bands at the VBM and CBM observed in the band structure is supported by the abrupt decrease to zero in the PDOS when analyzed in this region.

### Li Decoration

**Figure 4:**
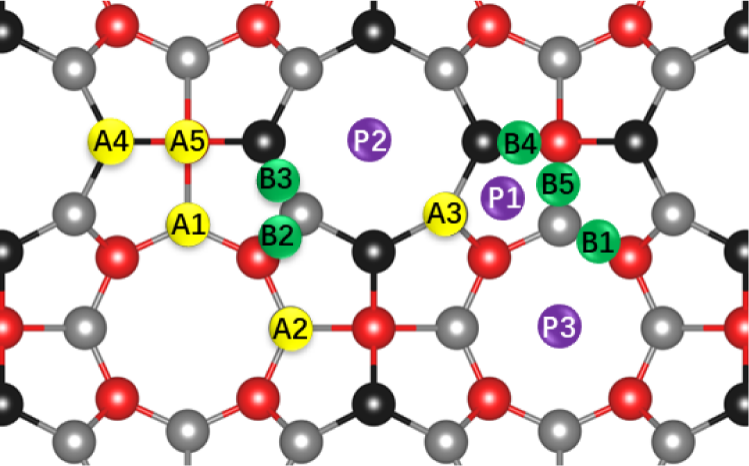
Adsorption sites evaluated for Li decoration on POG-B_4_C_2_N_3_.

Figure 4 illustrates the adsorption sites considered for Li decoration, which include 13 nonequivalent sites classified into five atom-centered sites (A), five bond-centered sites (B), and three pore-centered sites (P). The corresponding adsorption energies and final positions of the Li adatoms are summarized in Table 1. Upon analysis, it becomes evident that the Li adatoms consistently migrate to three energetically favorable sites: P2, A5, and P3, with adsorption energies of −3.69, −3.62, and −2.99 eV, respectively. In particular, the lower adsorption energies at A5 and P2 suggest that these sites exhibit a higher binding affinity, possibly due to optimal electronic interactions or geometric compatibility with Li. In contrast, the slightly less negative energy at P3 (−2.99 eV) could be associated with weaker coordination or steric effects. Our results represent a higher affinity for the Li adatom in POG-B_4_C_2_N_3_ than those found for the most stable site in the POG structure (−2.42 eV).^31^

**Table 1:** Adsorption Energies (*E*_ads_) and Final Configurations for the Adsorption Sites Evaluated during Li Decoration on the POG-B_4_C_2_N_3_ System

Initial site	*E*_ads_ (eV)	Final site
A1	–3.62	A5
A2	–3.69	P2
A3	–3.69	P2
A4	–3.69	P2
A5	–3.62	A5
B1	–3.62	A5
B2	–3.69	P2
B3	–3.69	P2
B4	–3.62	A5
B5	–3.62	A5
P1	–3.62	A5
P2	–3.69	P2
P3	–2.99	P3

**Figure 5:**
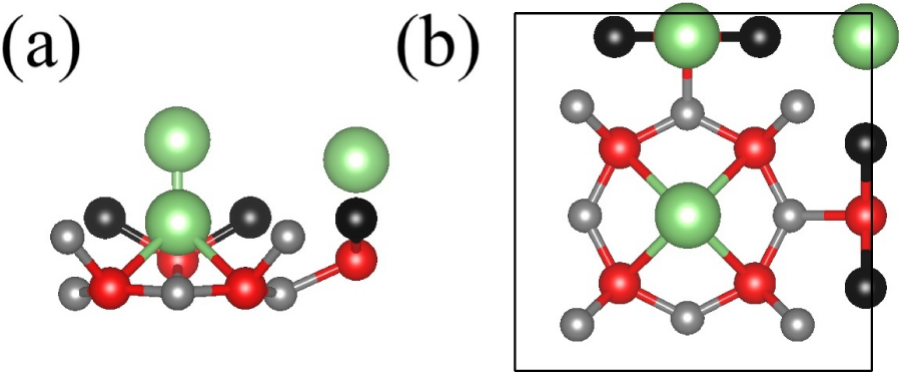
(a) Side and (b) top views of the final structure of Li@POG-B_4_C_2_N_3_ reaching 3 Li per unit cell.

**Figure 6:**
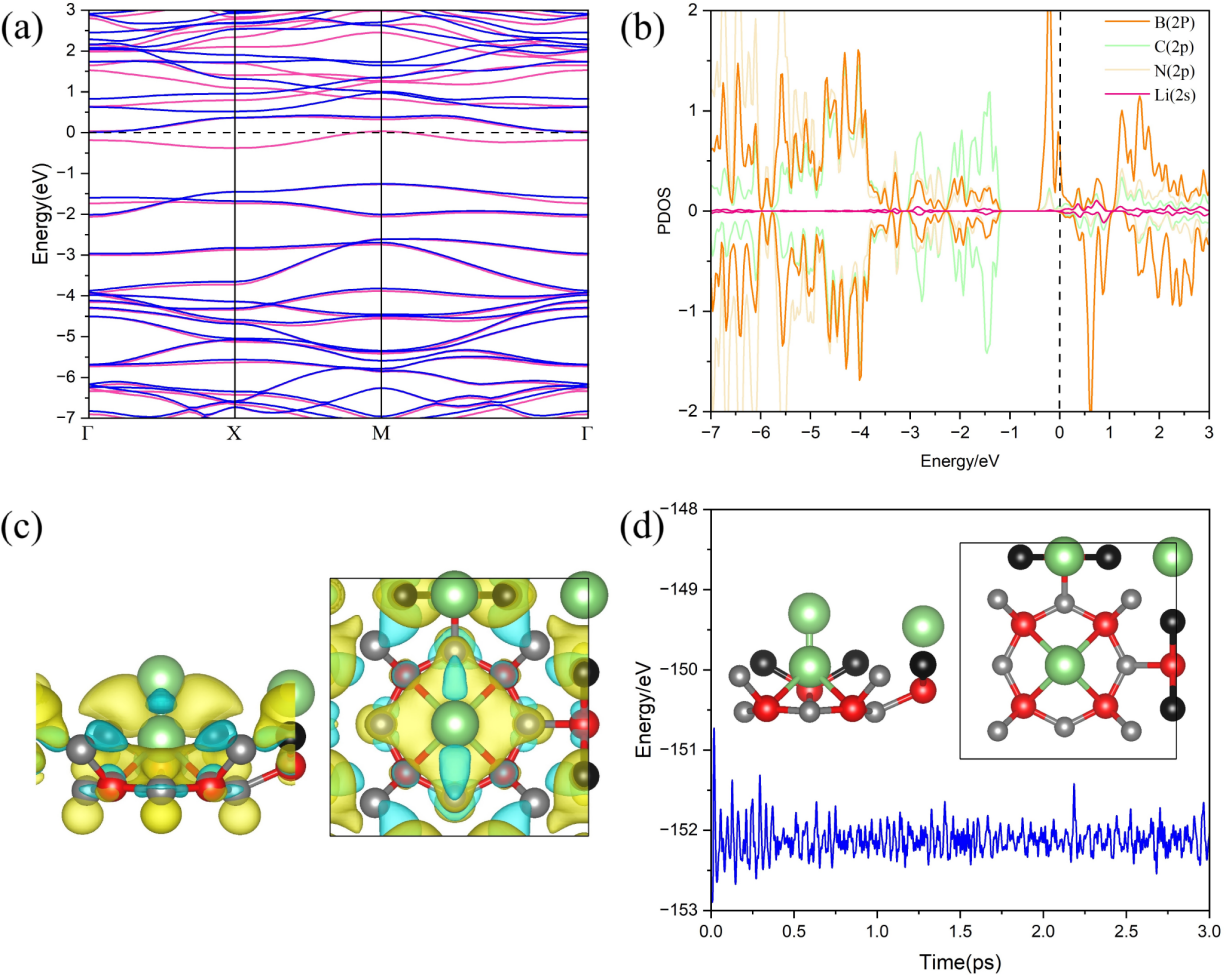
(a) Band structure, (b) partial density of states (PDOS), (c) charge density difference (CDD) map, and (d) energy fluctuations and final structure obtained from AIMD simulations at 300 K for the Li@POG-B_4_C_2_N_3_ system.

Based on the insights mentioned above, full Li saturation of POG-B_4_C_2_N_3_ was performed, as represented in Figure 5. The Li@POG-B_4_C_2_N_3_ system is achieved with a, Li coverage of 2 Li per unit cell. Figures 6a,b shows the spin-resolved band structure and partial density of states (PDOS) for the Li@B_4_C_2_N_3_ system. Both spin channels reveal metallic behavior with the Fermi level located at the first unoccupied band. In the α channel (red), a band is observed closer to the valence band maximum (VBM), shifted relative to the lower valence band states, and crosses the Fermi level at the M-point. Analyzing the PDOS, the state distribution is nearly identical to that of the pristine POG-B_4_C_2_N_3_ monolayer, with the exception of the additional Li states in the conduction band.

Figure 6c shows the charge density difference (CDD) map, where the blue and yellow regions indicate charge depletion and accumulation, respectively. For Li adsorbed at the octagonal pore, a strong charge accumulation is observed along the Li–N bond axis. Similarly, for Li adsorbed above the N atom, charge accumulation occurs along the C–Li bonds. Charge depletion is evident in the B atoms, suggesting that these atoms act as charge transfer centers for Li-POG-B_4_C_2_N_3_ bonds.

AIMD simulations were performed for the Li@POG-B_4_C_2_N_3_ system, as shown in Figure 6d. There are no reconstructions in the monolayer under thermal stimulus (300 K), which attests to good structural stability. Furthermore, the interaction between the Li adatoms and the substrate is sufficiently strong to keep the metals fixed within the structure. These results demonstrate the robustness and thermal stability of the Li@POG-B_4_C_2_N_3_ substrate, which are crucial for its potential use in hydrogen molecular adsorption.

### H_2_ Storage

**Figure 7:**
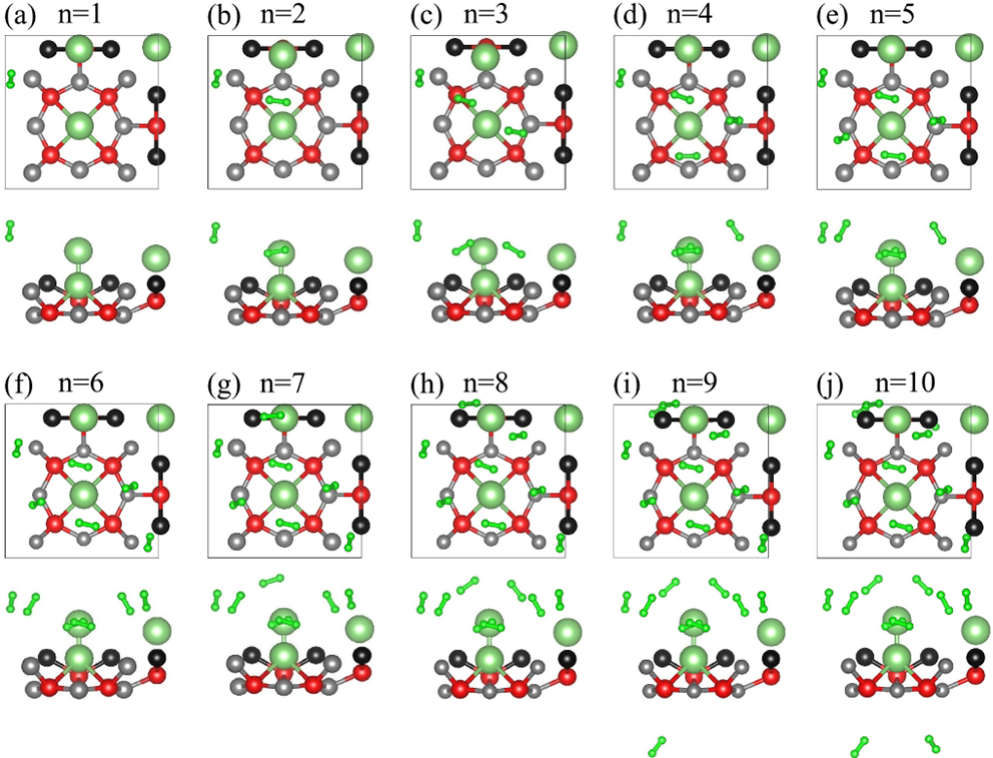
Optimized configurations of Li@POG-B_4_C_2_N_3_ with (a) 1H_2_, (b) 2H_2_, (c) 3H_2_, (d) 4H_2_, (e) 5H_2_, (f) 6H_2_, (g) 7H_2_, (h) 8H_2_, (i) 9H_2_, and (j) 10H_2_ molecules adsorbed.

Figure 7 illustrates the pathway used to achieve full adsorption of H_2_ coverage on the Li@POG-B_4_C_2_N_3_ system. Table 2 provides a comprehensive summary of (*E*_ads_) employing the D2 and D3 dispersion corrections, hydrogen adsorption capacity (HAC), average bond length of adsorbed H_2_ molecules (R_H–H_), and hydrogen release temperature (T_R_) for all adsorption models depicted in Figure 7.

**Table 2:** Adsorption Energy (*E*_ads_), Hydrogen Adsorption Capacity (HAC), Average H–H Bond Length (R_H–H_), and Release Temperature (*T*_R_) for Li@POG-B_4_C_2_N_3_ + nH_2_ (n = 1, 2, 3, 4, 5, 6, 7, 8, 9, and 10)

System	(eV)	(eV)	HAC (wt %)	R_H–H_(Å)	T_R_(K)
**Li@POG-B**_**4**_**C**_**2**_**N**_**3**_**+ 1H**_**2**_	–0.35	–0.30	0.84	0.79	444.94
**Li@POG-B**_**4**_**C**_**2**_**N**_**3**_**+ 2H**_**2**_	–0.35	–0.29	1.67	0.78	447.01
**Li@POG-B**_**4**_**C**_**2**_**N**_**3**_**+ 3H**_**2**_	–0.33	–0.25	2.50	0.78	422.06
**Li@POG-B**_**4**_**C**_**2**_**N**_**3**_**+ 4H**_**2**_	–0.26	–0.20	3.34	0.77	344.57
**Li@POG-B**_**4**_**C**_**2**_**N**_**3**_**+ 5H**_**2**_	–0.24	–0.22	4.18	0.77	312.19
**Li@POG-B**_**4**_**C**_**2**_**N**_**3**_**+ 6H**_**2**_	–0.24	–0.17	5.01	0.77	309.53
**Li@POG-B**_**4**_**C**_**2**_**N**_**3**_**+ 7H**_**2**_	–0.24	–0.15	5.84	0.77	308.85
**Li@POG-B**_**4**_**C**_**2**_**N**_**3**_**+ 8H**_**2**_	–0.22	–0.14	6.68	0.76	281.04
**Li@POG-B**_**4**_**C**_**2**_**N**_**3**_**+ 9H**_**2**_	–0.20	–0.14	7.52	0.76	261.19
**Li@POG-B**_**4**_**C**_**2**_**N**_**3**_ **+ 10H**_**2**_	–0.19	–0.13	8.35	0.76	245.29

The  () varies from −0.35 (−0.30) eV (Li@POG-B_4_C_2_N_3_ + 1H_2_) to −0.19 (−0.13) eV (Li@POG-B_4_C_2_N_3_ + 10H_2_) in the physisorption regime, very far away from the chemical adsorption noticed for the single Li adatom on the POG-B_4_C_2_N_3_ monolayer. This suggests that H_2_ molecules primarily interact with the Li@POG-B_4_C_2_N_3_ substrate via van der Waals forces, with no significant chemical bonding but changing the charge transfer mechanism between the Li metal and the monolayer. The adsorption energies of the D2 and D3 dispersion corrections consistently show that D2 is more negative, indicating stronger adsorption in D2. At lower hydrogen loadings (1–3 H_2_), the difference is small (0.05–0.08 eV). As more hydrogen molecules are adsorbed, the difference increases, especially beyond 4H_2_, with D3 showing weaker interactions. For example, at *n* = 4, the energies drop from −0.26 eV (D2) to −0.20 eV (D3) and at *n* = 10, from −0.19 eV (D2) to −0.13 eV (D3). This pattern occurs because D2 often overestimates dispersion due to its empirical nature, while D3′s environment-dependent corrections result in weaker binding. Increasing hydrogen loadings lead to weaker physisorption and suggest a saturation effect owing to steric and electronic factors.

Overall, although both methods follow the same qualitative trend, the D3 correction provides a more refined and often more accurate description of weak van der Waals interactions, making it a preferred choice for evaluating physisorption-dominated systems.

The HAC reaches an impressive 8.35 wt % for 10H_2_ molecules per unit cell, surpassing the minimum benchmark of 4.5 wt % set by the DOE for practical hydrogen storage materials. This result highlights the potential of Li@POG-B_4_C_2_N_3_ as a competitive candidate for hydrogen storage applications.

The H_2_ bond length (R_H–H_) remains nearly unchanged during the adsorption process, with a total variation of only 0.03 Å, indicating minimal distortion of the H_2_ molecules. This reinforces the observation that adsorption is primarily physical rather than chemical, which facilitates the desorption process during storage.

Regarding the hydrogen release temperature (*T*_R_), a decreasing trend is observed as the number of adsorbed H_2_ molecules increases. The maximum T_R_ is 444.94 K (Li@POG-B_4_C_2_N_3_ + 1H_2_), and the minimum is 245.29 K (Li@POG-B_4_C_2_N_3_ + 10H_2_). These values indicate a reversible hydrogen storage mechanism under near-room temperature conditions, which is a critical advantage for practical applications. Therefore, the Li@POG-B_4_C_2_N_3_ system presents fast desorption kinetics and favorable hydrogen reversibility for practical H_2_ storage.

The adsorption performance of Li@POG-B_4_C_2_N_3_ (present work) is compared with previously reported systems in Table 3. Our system adsorbs a total of 10 H_2_ molecules, which is lower than several other structures, such as Li@B_5_N_3_ (13), Li@net-Y (24), Li@irida-graphene (24), and Li@B_4_C_3_ (40). However, the absolute adsorption energy per H_2_ in our system (0.19 eV) is close to the values observed for Li@PdS_2_ (0.20 eV), Li@B_5_N_3_ (0.21 eV), and Li@B_4_C_3_ (0.23 eV), while it is higher than that of Li@IGP-SiC (0.14 eV).

**Table 3:** Total Number of Adsorbed H_2_ Molecules (n), Absolute Adsorption Energy Per H_2_ (|*E*_ads_|), Hydrogen Adsorption Capacity (HAC), and Desorption Temperature (T_des_) Associated with Configurations Exhibiting Complete H_2_ Coverage Configurations in Recently Documented Systems

System	n	|*E*_ads_| (eV)	HAC (wt %)	T_des_ (K)
Li@POG-B**_4_C_2_N_3_** (present work)	10	0.19	8.35	245
**Li@B**_**5**_**N**_**3**_(53)	13	0.21	6.30	267
**Li@net-Y**(54)	24	0.27	9.0	348
**Li@α-C**_**3**_**N**_**2**_(55)	12	0.22	5.7	285
**Li@B14**(56)	40	0.21	11.2	271
**Li@irida-graphene**(57)	24	0.28	7.06	353
**Li@IGP-SiC**(26)	48	0.14	8.27	191
**Li@C**_**4**_**N**(22)	24	0.28	8.00	358
**Li@B**_**4**_**C**_**3**_(58)	6	0.23	6.22	286
**Li@PdS**_**2**_(59)	5	0.20	6.98	-

Regarding the hydrogen adsorption capacity (HAC), our system achieves 8.35 wt %, surpassing Li@B_5_N_3_ (6.30 wt %), Li@α-C_3_N_2_ (5.7 wt %), and Li@PdS_2_ (6.98 wt %) but remaining slightly lower than Li@C_4_N (8.00 wt %) and Li@net-Y (9.0 wt %). In contrast, Li@B_4_C_3_ exhibits the highest HAC (11.2 wt %).

Finally, the desorption temperature (T_des_) of 245 K for Li@POG-B_4_C_2_N_3_ is relatively moderate, higher than that of Li@IGP-SiC (191 K) but lower than that of Li@C_4_N (358 K), Li@irida-graphene (353 K), and Li@net-Y (348 K). This suggests that our system balances the adsorption strength and thermal stability, making it a competitive candidate for hydrogen storage applications.

**Figure 8:**
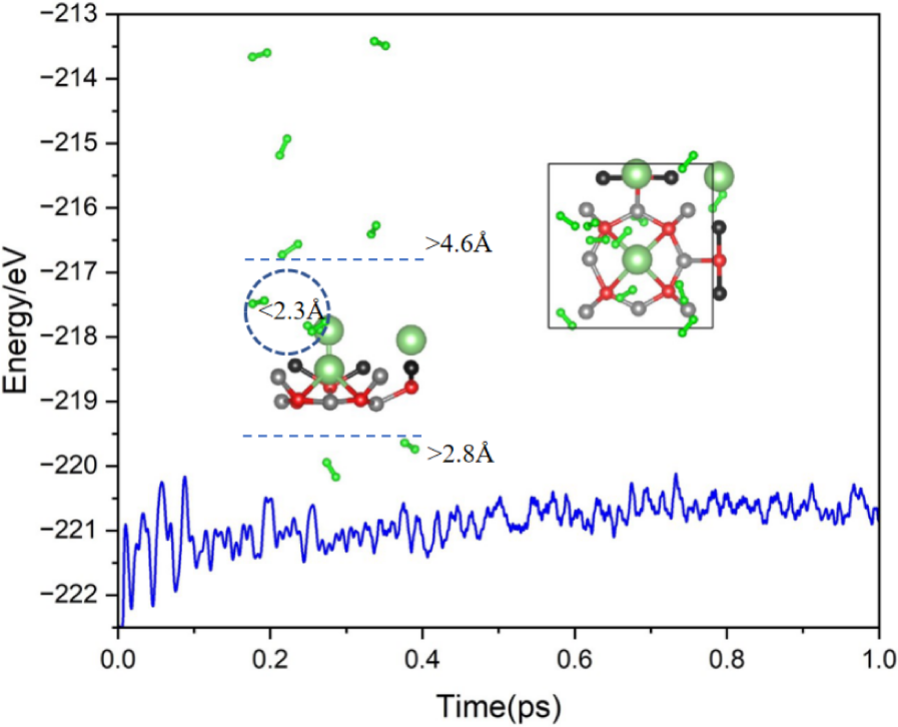
Energy fluctuations and the final structure obtained from AIMD simulations at 300 K for the Li@POG-B_4_C_2_N_3_ + 10H_2_ system.

Figure 8 presents the results of the AIMD simulations for the Li@POG-B_4_C_2_N_3_ + 10H_2_ system at 300 K over a simulation time of 1.0 ps, together with the final structure of the system. The results reveal that most H_2_ molecules rapidly desorb from the substrate, consistent with the low T_R_ = 245.29 K reported in Table 2. This behavior highlights the system’s potential for efficient and rapid hydrogen release under near-ambient conditions. The simulation further indicates that the Li@POG-B_4_C_2_N_3_ substrate retains its structural integrity with minimal distortion during the desorption process. Notably, the Li atom remains adsorbed securely on the substrate, demonstrating the stability of the active site, even under dynamic conditions. This stability ensures the substrate’s reusability for multiple adsorption–desorption cycles. Energy fluctuations during the simulation exhibit an initial variation around 2 eV, which stabilizes to a fluctuation range below 1 eV after approximately 0.5 ps. This stabilization reflects the system’s transition to a thermodynamically stable state after the desorption of H_2_ molecules. Therefore, AIMD simulations confirm the fast H_2_ release in a short simulation time and the feasibility of the Li@POG-B_4_C_2_N_3_ system in practical use.

**Figure 9:**
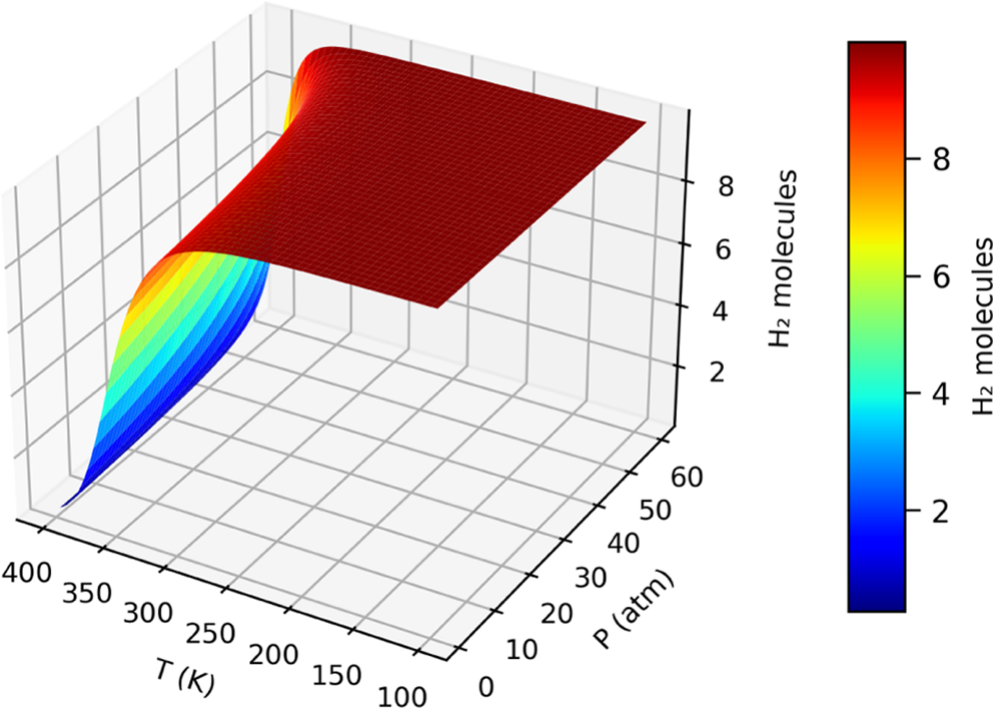
Number of H_2_ molecules adsorbed on Li@POG-B_4_C_2_N_3_ with varying pressure (P) and temperature (T).

Furthermore, we investigated how varying thermodynamic conditions affect the hydrogen storage characteristics of Li@POG-B_4_C_2_N_3_ by simulating the occupancy of the H_2_ molecule at different temperature (T) and pressure (P) levels, as shown in Figure 9. Li@POG-B_4_C_2_N_3_ can host 10 H2 molecules (8.35 wt %) at 325 K and pressures exceeding 10 bar, maintaining this capacity consistently over a broad range of temperatures and pressures, which underscores the robustness of hydrogen storage in this new material.

**Figure 10:**
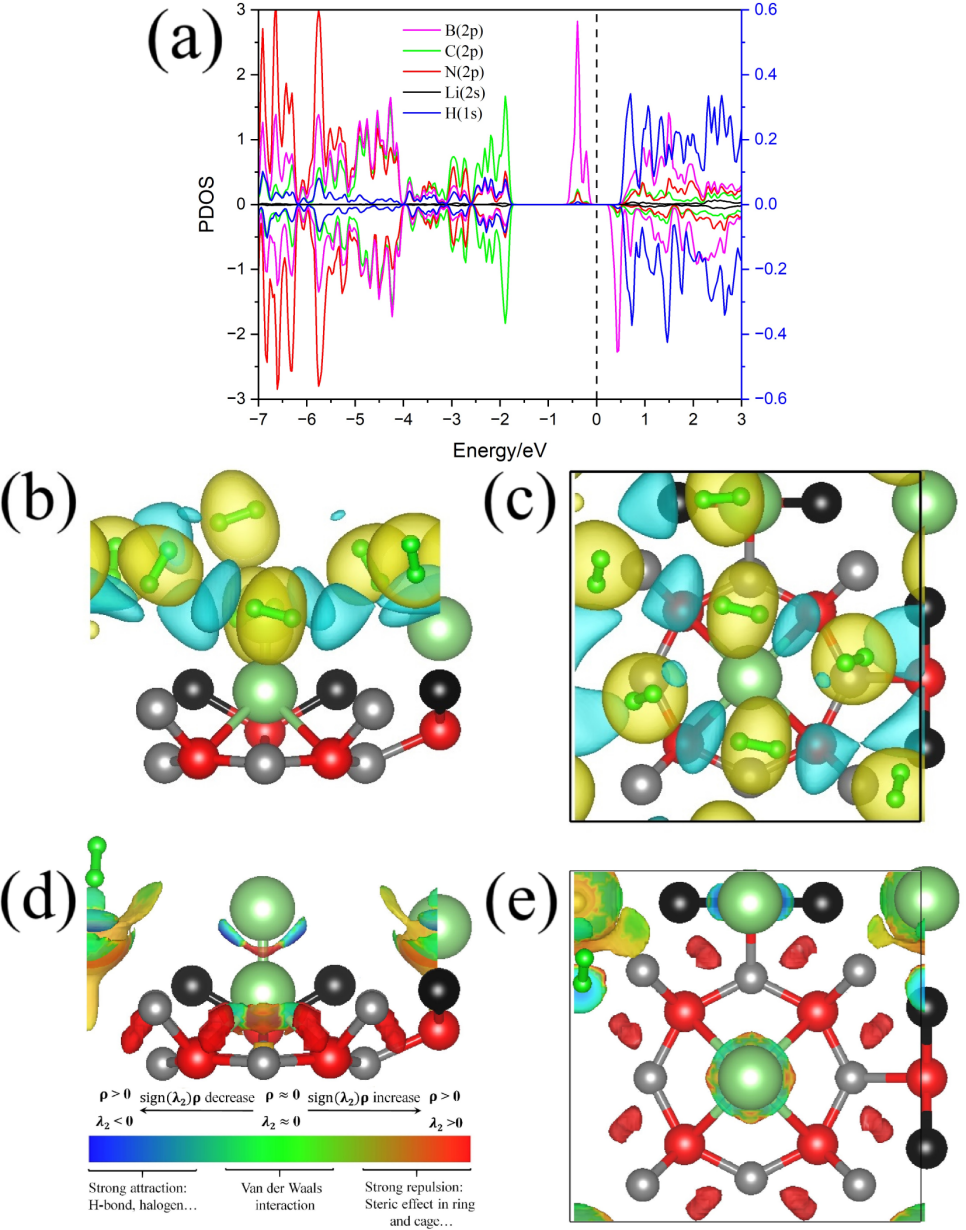
(a) Partial density of states (PDOS), (b) side and (c) top views of charge density difference (CDD) map, and (d) side and (e) top views of reduced density gradient (RDG) for the Li@POG-B_4_C_2_N_3_ + 10H_2_ system.

To investigate the influence of H_2_ molecules on the electronic properties of the Li@POG-B_4_C_2_N_3_ system, the spin-resolved PDOS for Li@POG-B_4_C_2_N_3_ + 10H_2_ is shown in Figure 10. The analysis reveals a significant bandgap opening of approximately 0.65 eV, considering the states present in both spin channels. This transition from metallic to semiconducting behavior highlights the critical role of H_2_ molecules in modulating the electronic structure of the system, suggesting the viability of Li-decorated POG-B_4_C_2_N_3_ as a potential H_2_ sensor.

A detailed examination of PDOS further reveals notable changes in the electronic states near the band edges. At the valence band maximum (VBM), there is an increased contribution from boron B states, which contrasts with the pristine and Li-decorated POG-B_4_C_2_N_3_ nanosheet, where carbon C states predominate in this region. Similarly, the conduction band minimum (CBM) for both spin channels exhibits a strong presence of B states. Across the energy range evaluated for the conduction band (CB), H_2_ states consistently present relevant contributions, indicating a significant hybridization between the states of the adsorbed hydrogen molecules and the substrate.

The interactions within the Li@POG-B_4_C_2_N_3_ + 10H_2_ system were analyzed by using the reduced density gradient (RDG) method to characterize the nature of these interactions. Furthermore, the charge density difference (CDD) isosurface was used to assess changes in the electronic environment resulting from the adsorption of 10H_2_ molecules on Li@POG-B_4_C_2_N_3_ (Figures 10b,c).

The CDD map reveals charge accumulation along the H_2_ molecules and indicates a minor charge transfer from the Li@POG-B_4_C_2_N_3_ substrate to the adsorbed H_2_ molecules. The absence of a significant charge density between Li and H_2_ supports the weak nature of their interaction. Further analysis via RDG reveals that λ_2_ values along the H_2_–Li bond approach zero, confirming that weak van der Waals forces predominantly govern the interaction. This aligns with the observed low adsorption energy values, making the system suitable for efficient H_2_ storage. Interestingly, the interactions between Li and the POG-B_4_C_2_N_3_ substrate are more complex. Along the bond axes, λ_2_ is >0, highlighting a strong steric influence. Despite this, Li adatoms form strong lateral covalent bonds with the B atoms.

## Conclusions

In this study, we introduced a novel inorganic analogue of POG, namely, POG-B_4_C_2_N_3_, through density functional theory (DFT). The stability of this compound was demonstrated using several key descriptors, including cohesive energy, phonon dispersion, Born–Huang criteria, and ab initio molecular dynamics (AIMD) simulations. The AIMD analysis at 300 K confirmed the structural integrity of the monolayer, with no significant distortions or reconstructions observed, confirming its thermal stability.

POG-B_4_C_2_N_3_ was identified as a narrow band gap semiconductor with a direct band gap of 0.86 eV at the HSE level, while the PBE functional provided a gap of 0.32 eV. Its mechanical properties further reinforce its stability, with elastic constants satisfying the Born–Huang criteria for a square lattice. The anisotropy in the Poisson ratio (ν), with maximum and minimum values of 0.32 and −0.05, respectively, classifies POG-B_4_C_2_N_3_ as an auxetic material.

Additionally, we explored the Li decoration of POG-B_4_C_2_N_3_ to evaluate its potential for hydrogen storage. Remarkably, the hydrogen adsorption capacity (HAC) reached 8.35 wt % for 10 H_2_ molecules per unit cell, significantly surpassing the Department of Energy’s benchmark of 4.5 wt % for practical hydrogen storage materials. The study also analyzed the hydrogen storage performance of Li@POG-B_4_C_2_N_3_ under varying thermodynamic conditions, showing consistent storage capacities over a broad range of temperatures and pressures. For example, at 325 K and pressures exceeding 10 bar, the system maintained a capacity of 10 H_2_ molecules (8.35 wt %). This robustness under practical conditions further emphasizes the potential of this material for hydrogen storage applications.

In summary, POG-B_4_C_2_N_3_ emerges as a mechanically stable and thermally robust monolayer with promising semiconducting properties, high hydrogen storage capacity, minimal structural distortions on the substrate, and reversible adsorption–desorption dynamics, making it a promising candidate for real-world hydrogen storage technologies.

## Data Availability

Data supporting the results can be accessed by contacting the corresponding author.
